# Renoprotective Effects of Alpha-1 Antitrypsin against Tacrolimus-Induced Renal Injury

**DOI:** 10.3390/ijms21228628

**Published:** 2020-11-16

**Authors:** Jeong-Hoon Lim, Eun-Joo Oh, Se-Hyun Oh, Hee-Yeon Jung, Ji-Young Choi, Jang-Hee Cho, Sun-Hee Park, Yong-Lim Kim, Chan-Duck Kim

**Affiliations:** Division of Nephrology, Department of Internal Medicine, School of Medicine, Kyungpook National University, Kyungpook National University Hospital, Daegu 41944, Korea; jh-lim@knu.ac.kr (J.-H.L.); oej1124@naver.com (E.-J.O.); ttily@nate.com (S.-H.O.); hy-jung@knu.ac.kr (H.-Y.J.); jyss1002@hanmail.net (J.-Y.C.); jh-cho@knu.ac.kr (J.-H.C.); sh-park@knu.ac.kr (S.-H.P.); ylkim@knu.ac.kr (Y.-L.K.)

**Keywords:** alpha-1 antitrypsin, tacrolimus-induced renal injury, fibrosis, inflammation, apoptosis

## Abstract

The protective effects of alpha-1 antitrypsin (AAT) in tacrolimus (TAC)-induced renal injury was evaluated in a rat model. The TAC group rats were subcutaneously injected with 2 mg/kg TAC every day for four weeks. The TAC with AAT group was cotreated with daily subcutaneous injections of TAC and intraperitoneal injections of AAT (80 mg/kg) for four weeks. The effects of AAT on TAC-induced renal injury were evaluated using serum biochemistry, histopathology, and Western blotting. The TAC injection significantly increased renal interstitial fibrosis, inflammation, and apoptosis as compared to the control treatment. The histopathological examination showed that cotreatment of TAC and AAT attenuated interstitial fibrosis (collagen, fibronectin, and α-SMA staining), and α-SMA expression in Western blotting was also decreased. Immunohistochemical staining for inflammation (osteopontin and ED-1 staining) revealed improved interstitial inflammation in the TAC with AAT group compared to that in the TAC group. The TAC treatment increased renal apoptosis compared to the control treatment, based on the results of increased immunohistochemical staining of terminal deoxynucleotidyl transferase-mediated dUTP nick-end labeling (TUNEL), increased caspase-3 activity, and lower Bcl-2 to Bad expression ratio. However, AAT cotreatment significantly changed these markers and consequently showed decreased apoptosis. AAT protects against TAC-induced renal injury via antifibrotic, anti-inflammatory, and antiapoptotic effects.

## 1. Introduction

The potent calcineurin inhibitor, tacrolimus (TAC), was introduced in 1984. Currently, the TAC-based immunosuppressive regimen is the globally preferred treatment in kidney transplant (KT) recipients [1,2]. However, despite the effective immunosuppressive property, long-term TAC treatment causes irreversible kidney injury and adversely affects long-term graft survival [3]. The histopathologic features of TAC-induced renal injury are arteriolar hyalinization, vasoconstriction, interstitial fibrosis (IF), tubular atrophy, and apoptosis [4,5,6]. The pathophysiologic mechanism remains unclear; however, several in vivo and in vitro studies have identified that inflammatory mediates and apoptosis are linked to TAC-induced nephropathy [7,8,9]. 

Alpha-1 antitrypsin (AAT) is a member of the serine protease inhibitor superfamily and can inhibit other proteases, such as cysteine aspartic proteases [10,11]. AAT has a broad range of inhibitory functions, including anti-inflammatory properties and, as a result, can limit tissue injury resulting from proteases [12]. Moreover, AAT protects against apoptosis in various types of cells, such as peritoneal mesenchymal cells, beta cells, and hepatocytes [13,14,15]. In particular, it is known that the AAT’s inhibitory function of cysteine proteases of the caspase family, such as caspase-3, plays an important role in preventing apoptosis [15].

Based on these findings, we have assumed that the anti-inflammatory effect and antiapoptotic function of AAT can attenuate TAC-induced nephrotoxicity. Thus, the present study was designed with the aim of investigating the renoprotective effects of AAT using an experimental rat model for TAC-induced renal injury.

## 2. Results

### 2.1. Effect of AAT on the Basic Parameters

Table 1 shows the basic parameters. After 5 weeks of treatment, the TAC treatment caused a significant decrease in weight gain as compared to the vehicle (VH) treatment (TAC vs. VH; *p* < 0.001). Weight gain in the TAC-treated rats was unaffected by AAT (TAC vs. TAC + AAT; *p* > 0.05). The serum blood urea nitrogen (BUN) and creatinine levels were higher in the TAC and TAC + AAT groups as compared to that in the VH group (TAC vs. TAC + AAT; both *p* < 0.001). Concomitantly administered AAT with TAC decreased the serum BUN and creatinine as compared to the TAC treatment (TAC vs. TAC + AAT; both *p* < 0.01). The measured serum TAC trough levels after 5 weeks were similar between the TAC and TAC + AAT groups (TAC vs. TAC + AAT; *p* > 0.05), indicating that AAT did not affect the TAC concentration.

### 2.2. Effect of AAT on Renal Fibrosis in TAC-Induced Renal Injury

**Figure 1:**
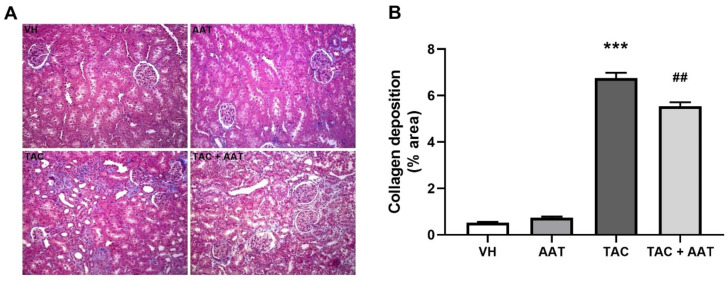
Effect of alpha-1 antitrypsin on collagen deposition in tacrolimus-induced renal injury. (**A**) Trichrome staining (200× magnification). (**B**) Quantitative analysis of collagen deposition area. Data are presented as means ± standard errors; *** *p* < 0.001 vs. VH; ^##^ *p* < 0.01 vs. TAC. Abbreviations: VH, vehicle group; AAT, alpha-1 antitrypsin treatment group; TAC, tacrolimus treatment group; TAC + AAT, tacrolimus and alpha-1 antitrypsin cotreatment group.

**Figure 2:**
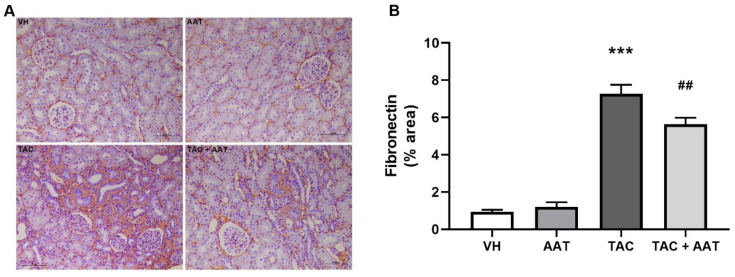
Effect of alpha-1 antitrypsin on fibronectin staining in tacrolimus-induced renal injury. (**A**) Immunohistochemical staining for fibronectin (200× magnification). (**B**) Quantitative analysis of fibronectin staining area. Data are presented as means ± standard errors; *** *p* < 0.001 vs. VH; ^##^ *p* < 0.01 vs. TAC. Abbreviations: VH, vehicle group; AAT, alpha-1 antitrypsin treatment group; TAC, tacrolimus treatment group; TAC + AAT, tacrolimus and alpha-1 antitrypsin cotreatment group.

**Figure 3:**
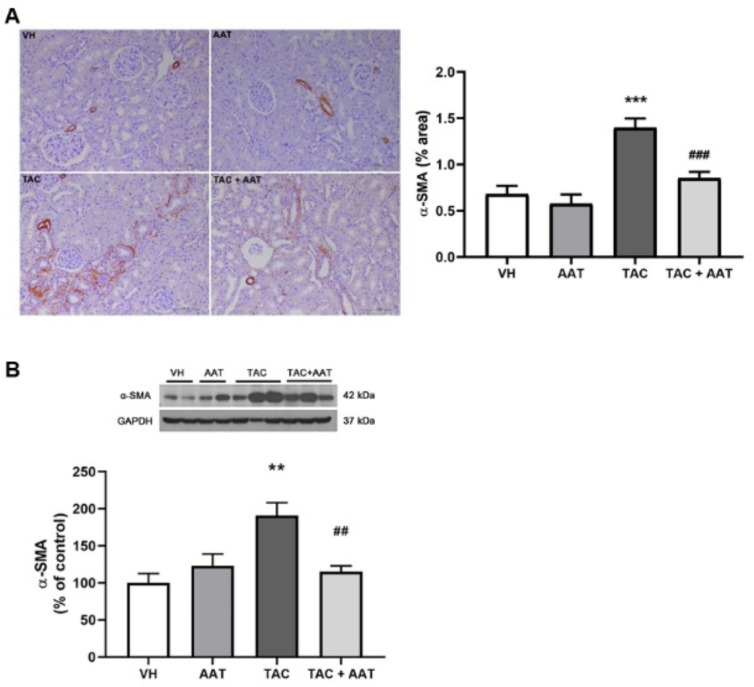
Effect of alpha-1 antitrypsin on α-SMA staining and expression in tacrolimus-induced renal injury. (**A**) Immunohistochemical staining for α-SMA (200× magnification) (Left). Quantitative analysis of α-SMA staining area (Right). (**B**) Immunoblotting analysis of α-SMA. Data are presented as means ± standard errors; ** *p* < 0.01 vs. VH; *** *p* < 0.001 vs. VH; ^##^ *p* < 0.01 vs. TAC; ^###^ *p* < 0.001 vs. TAC. Abbreviations: VH, vehicle group; AAT, alpha-1 antitrypsin treatment group; TAC, tacrolimus treatment group; TAC + AAT, tacrolimus and alpha-1 antitrypsin cotreatment group.

In the comparison of histopathology, the TAC group showed significantly increased collagen deposition in the trichrome stain compared to the VH group (TAC vs. VH; *p* < 0.001, Figure 1); however, cotreatment of TAC and AAT reduced collagen deposition compared to TAC (TAC vs. TAC + AAT; *p* < 0.01). Immunohistochemical staining for fibrosis markers, fibronectin (Figure 2), and α-SMA (Figure 3A) showed increased staining in the TAC group as compared to that in the VH group (TAC vs. VH; both *p* < 0.001) and the stained degree of fibronectin and α-SMA were reduced in the cotreated TAC and AAT group as compared to that in the TAC group (TAC vs. TAC + AAT; *p* < 0.01 and *p* < 0.001, respectively).

Further, Western blotting analysis of the kidneys revealed a significant increase in the α-SMA expression in the TAC group over the VH group (TAC vs. VH; *p* < 0.01); however, the α-SMA expression was decreased in the group treated with TAC and AAT together as compared to the group treated with TAC (TAC vs. TAC + AAT; *p* < 0.01) (Figure 3B).

### 2.3. Effect of AAT on Inflammation in TAC-Induced Renal Injury

**Figure 4:**
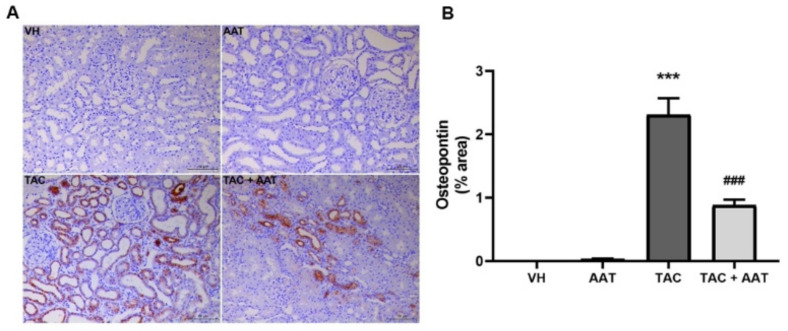
Effect of alpha-1 antitrypsin on osteopontin staining in tacrolimus-induced renal injury. (**A**) Immunohistochemical staining for osteopontin (200× magnification). (**B**) Quantitative analysis of osteopontin staining area. Data are presented as means ± standard errors; *** *p* < 0.001 vs. VH; ### *p* < 0.001 vs. TAC. Abbreviations: VH, vehicle group; AAT, alpha-1 antitrypsin treatment group; TAC, tacrolimus treatment group; TAC + AAT, tacrolimus and alpha-1 antitrypsin cotreatment group.

**Figure 5:**
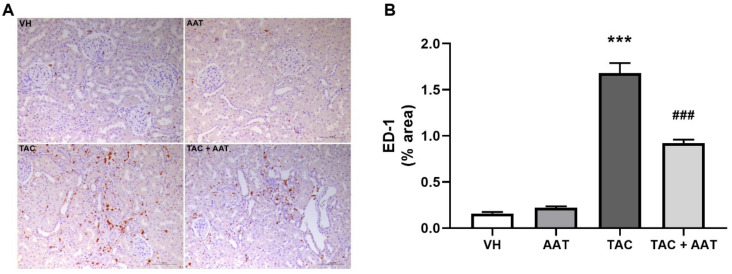
Effect of alpha-1 antitrypsin on ED-1 staining in tacrolimus-induced renal injury. (**A**) Immunohistochemical staining for ED-1 (200× magnification). (**B**) Quantitative analysis of ED-1 staining area. Data are presented as means ± standard errors; *** *p* < 0.001 vs. VH; ^###^ *p* < 0.001 vs. TAC. Abbreviations: VH, vehicle group; AAT, alpha-1 antitrypsin treatment group; TAC, tacrolimus treatment group; TAC + AAT, tacrolimus and alpha-1 antitrypsin cotreatment group.

Immunohistochemical staining for OPN and ED-1 was used to evaluate the changes of inflammation. OPN expression was dramatically increased in the TAC group as compared to that in the VH group (TAC vs. VH; *p* < 0.001). In particular, OPN was stained in areas with active inflammation (Figure 4). The TAC and AAT cotreatment group revealed a significant reduction in the OPN expression as compared to the TAC group. There were few ED-1 positive cells in the VH group; however, the number was significantly increased after TAC treatment (TAC vs. VH; *p* < 0.001) (Figure 5). Cotreatment with AAT decreased the number of ED-1 positive cells as compared to TAC treatment alone (TAC vs. TAC + AAT; *p* < 0.001).

### 2.4. Effect of AAT on Apoptosis in TAC-Induced Renal Injury

**Figure 6:**
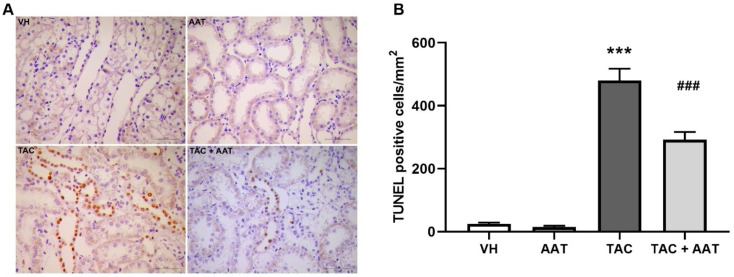
Effect of alpha-1 antitrypsin on apoptosis detected by terminal deoxynucleotidyl transferase-mediated dUTP nick-end labeling (TUNEL) assay in tacrolimus-induced renal injury. (**A**) Immunohistochemical staining for TUNEL staining (400× magnification). (**B**) Quantitative analysis of TUNEL-positive cells. Data are presented as means ± standard errors; *** *p* < 0.001 vs. VH; ^###^ *p* < 0.001 vs. TAC. Abbreviations: VH, vehicle group; AAT, alpha-1 antitrypsin treatment group; TAC, tacrolimus treatment group; TAC + AAT, tacrolimus and alpha-1 antitrypsin cotreatment group.

**Figure 7:**
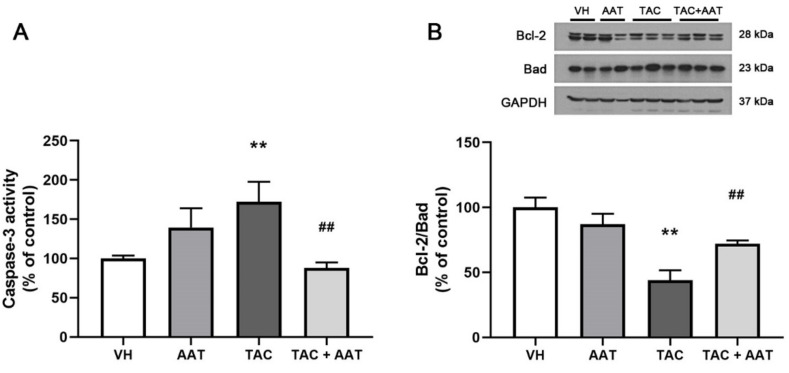
(**A**) Effect of alpha-1 antitrypsin on caspase-3 activity, and (**B**) Bcl-2 and Bad protein expression in tacrolimus-induced renal injury. Data are presented as means ± standard errors; ** *p* < 0.01 vs. VH; ^##^ *p* < 0.01 vs. TAC. Abbreviations: VH, vehicle group; AAT, alpha-1 antitrypsin treatment group; TAC, tacrolimus treatment group; TAC + AAT, tacrolimus and alpha-1 antitrypsin cotreatment group.

In order to evaluate the antiapoptotic effect of AAT in TAC-induced renal injury, we performed the terminal deoxynucleotidyl transferase-mediated dUTP nick-end labeling (TUNEL) assay, measured the caspase-3 activity, and determined the Bcl-2/Bad expressions. In the immunohistochemical staining, there were significantly more TUNEL-positive cells in the TAC group than in the VH group (TAC vs. VH; *p* < 0.001), and apoptosis was decreased with AAT cotreatment (TAC vs. TAC + AAT; *p* < 0.001) (Figure 6). Caspase-3 activity was significantly higher in the TAC group than in the VH group (TAC vs. VH; *p* < 0.01), while it was decreased in the AAT cotreated group as compared to that in the TAC-treated group (TAC vs. TAC + AAT; *p* < 0.01) (Figure 7A). We also conducted immunoblotting analysis of Bcl-2 and Bad expression to identify the antiapoptotic pathway. The Bcl-2 to Bad ratio was lower in the TAC group as compared to that in the VH group (TAC vs. VH; *p* < 0.01), however, the ratio was higher in the AAT cotreated group than in the TAC-treated group (TAC vs. TAC + AAT; *p* < 0.01) (Figure 7B).

## 3. Discussion

In this study, we have demonstrated the protective effect of AAT against TAC-induced renal injury. AAT exhibited anti-inflammatory and antiapoptotic effects in TAC-induced nephrotoxicity; AAT treatment also reduced renal fibrosis. Our results suggest that AAT treatment in patients with long-term medication of TAC, such as KT recipients, can prevent and reduce chronic TAC nephrotoxicity.

TAC is the most widely using calcineurin inhibitor in KT recipients [2]. However, chronic TAC nephropathy caused by the long-term use of TAC is a major cause of late graft failure in KT recipients [16]. Therefore, researchers are interested in reducing nephrotoxicity, and several trials have been conducted to inhibit the various pathophysiologic pathways involved in TAC-induced nephrotoxicity [3,17,18]. We have focused on the effects of AAT on renal fibrosis, inflammation, and apoptosis. AAT plays an important role not only in protease inhibition, but also in inflammation inhibition and tissue protection [13]. AAT modulates both the activation and maturation of antigen-presenting cells [19,20,21], inhibition of various caspases, and improvement of the mitochondrial membrane stability [22]. Thus, AAT downregulates proinflammatory cytokines and upregulates anti-inflammatory mediators [19,21,23], with these properties, AAT attenuates tissue fibrosis and prevents apoptotic cell death [19,24,25,26]. The function of AAT has not yet been identified in TAC-induced nephrotoxicity; however, it has been verified in the present study.

The patchy striped pattern of IF is a common feature of chronic TAC nephrotoxicity because TAC-induced nephrotoxicity preferentially involves the medullary rays [27]. The IF is a structural hallmark with collagen deposition that correlates with progressive and chronic kidney disease; further, it is a more consistent predictor of renal function impairment than glomerular injury [27,28,29]. We demonstrated the antifibrotic effect of AAT in TAC-induced renal injury through histopathology and immunoblotting assay. Fibronectin is a major extracellular matrix protein that acts as a chemoattractant of fibroblasts [30]; α-SMA is used as a fibrogenic activity indicator of activated tissue fibrogenic cells [31,32]. The results are consistent with our previous studies that have confirmed the antifibrotic effect of AAT in unilateral ureter obstruction and renal ischemia reperfusion injury models [33,34]. In our previous study, we have identified the relevant antifibrotic pathway of AAT treatment, that is, the suppression of the TGF-β/Smad3 signaling pathway [33]; the AAT’s antifibrotic effect in TAC nephropathy would also have been achieved via the inhibition of the TGF-β/Smad3 signaling pathway.

TAC treatment causes inflammatory cell infiltration and the production of proinflammatory cytokines in the kidneys [9]. TAC treatment-induced inflammation causes the development and amplification of fibrosis [35]. AAT exerts anti-inflammatory effects by suppressing neutrophil chemokine release and reducing superoxide production by neutrophils [36,37]. In order to evaluate the anti-inflammatory effects of AAT on TAC-induced renal injury, we identified two inflammatory markers (OPN and ED-1). OPN is known to possess cell adhesive and migratory properties [38,39]. In particular, OPN binds strongly to macrophages, resulting in prominent macrophage infiltration [40,41]. In the in vitro and in vivo TAC nephropathy studies, tubular OPN expression was avidly associated with macrophage infiltration and subsequent tubulointerstitial injury [3,42]. Our results were also consistent with these findings; OPN was overexpressed in the TAC group, notably in the inflammatory areas. However, the cotreatment of TAC and AAT lowered the OPN expression, supporting the reduction in inflammation with AAT treatment. Moreover, ED-1 is a well-known pan-macrophage marker [43]; TAC treatment induced increased ED-1 staining; however, ED-1 staining was decreased with AAT cotreatment. These results show that reduced infiltration of macrophages after AAT treatment and that the anti-inflammatory effects of AAT contribute toward the protection from TAC-induced renal injury.

Apoptosis is a process of programmed cell death; unlike necrosis, apoptosis does not involve inflammation. TAC induces dose- and time-dependent renal cell apoptosis, and apoptosis plays an important role in TAC-induced renal injury [44,45,46]. We also confirmed the antiapoptotic effect of AAT aside from antifibrotic and anti-inflammatory effect. Caspase-3 is known to be a crucial mediator of apoptosis owing to its catalyzing role in many key cellular proteins [15]. AAT directly binds to caspase-3 and inhibits the activity [47]. In our results, AAT also inhibited caspase-3 activity, and caused decreased apoptosis in TAC-induced renal injury. Activation of the mitochondrial pathway has been identified as the main apoptotic pathway in TAC-induced renal injury [44]. In order to clearly confirm the antiapoptotic effect in TAC-induced apoptosis, we evaluated the Bcl-2 and Bad protein expressions. Both, Bcl-2 and Bad are members of the Bcl-2 family; they play the opposite roles of antiapoptosis (Bcl-2) and proapoptosis (Bad), in particular, they control the intrinsic (mitochondrial) apoptosis at the upstream of the apoptotic pathway [48,49]. Further, our results showed a lower ratio of Bcl-2 to Bad expression in the TAC-treated group than in the VH group; however, the ratio was increased in the AAT cotreated group as compared to that in the TAC group. This suggests that AAT prevents mitochondria-mediated intrinsic apoptotic pathway in chronic TAC nephrotoxicity.

The present study confirmed that AAT treatment prevented chronic TAC nephrotoxicity in an experimental rat model. The renoprotective effects of AAT in TAC-induced nephrotoxicity were manifested as renal fibrosis, inflammation, and apoptosis, especially, AAT would directly attenuate tubulointerstitial inflammation and renal cell apoptosis. Because of the multifactorial benefits, AAT treatment is expected to improve the long-term graft survival in KT recipients. However, there are certain limitations of this study. The experimental model for TAC-induced nephrotoxicity was established in rats. Further, it has been reported that asymptomatic healthy individuals also have decreased AAT levels [50]; therefore, the effect of AAT in human subjects is unclear. Moreover, we did not measure the functionally active AAT levels. The presence of the inactivated forms of AAT has been reported previously [51] and might attenuate the effect of AAT. In addition, we did not evaluate the effect of post-administration of AAT. Well-designed clinical studies are needed to verify the effectiveness of AAT in human subjects.

In conclusion, AAT attenuates TAC-induced renal injury in an experimental rat model via antifibrotic, anti-inflammatory, and antiapoptotic effects. Our results demonstrate the therapeutic potential of AAT in protecting against chronic TAC nephrotoxicity.

## 4. Materials and Methods

### 4.1. Experimental Design

**Figure 8:**
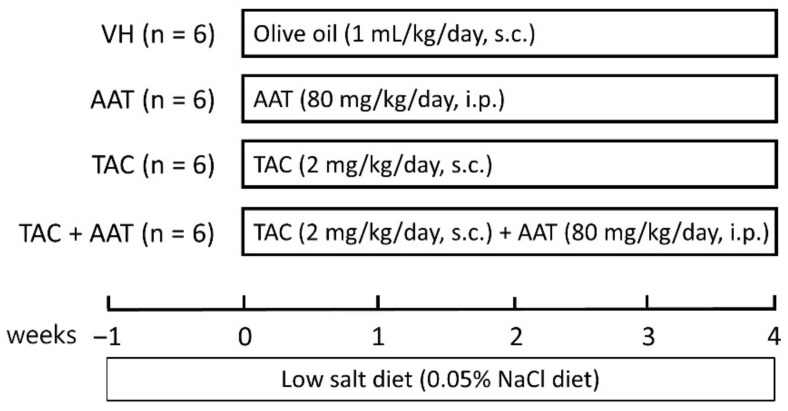
Schematic diagram of the study design. Abbreviations: VH, vehicle group; AAT, alpha-1 antitrypsin treatment group; TAC, tacrolimus treatment group; TAC + AAT, tacrolimus and alpha-1 antitrypsin cotreatment group; s.c., subcutaneous injection; i.p., intraperitoneal injection.

Eight-week-old male Sprague–Dawley rats (Hyochang Science, Daegu, South Korea), initial weight 220–230 g, were sheltered in a facility free of pathogens with a 12-h light/dark schedule, and they had free access to food and water at the animal care facility of Kyungpook National University. The animal experiment protocol was approved by the Animal Care and Use Committee at the Kyungpook National University (KNU-2019-0007; approval date: 9/1/2019), and all experiments on animal performed in this study were based on ethical guidelines for animal studies. The schematic experimental design is shown in Figure 8. The rats were fed with a low-salt diet (0.05% sodium, Teklad Premier, Madison, WI, USA). Tacrolimus (CKD Bio Corporation, Seoul, South Korea) was dissolved in olive oil (Sigma-Aldrich, St. Louis, MO, USA) to the final concentration of 1 mg/mL. After acclimatization and a low-salt diet for 1 week, the rats were randomized into 4 groups and were treated daily for 4 weeks as follows: subcutaneous injection with olive oil (1 mg/mL) (VH group; *n* = 6); intraperitoneal injection with AAT (80 mg/kg) (AAT group; Aralast; Baxter Healthcare, Vienna, Austria; *n* = 6); subcutaneous injection with TAC (2 mg/kg) (TAC group; *n* = 6); and simultaneous treatment with TAC and AAT (TAC + AAT group; *n* = 6).

### 4.2. Quantification of Tacrolimus

Serum tacrolimus concentrations were measured using a commercially available enzyme-linked immunosorbent assay kit (MyBiosource, San Diego, CA, USA) as per the manufacturer’s instructions. Values were calculated using a standard curve.

### 4.3. Renal Function and Histopathological Studies

The serum levels of BUN and creatinine were measured using a Hitachi 7600 automated biochemistry analyzer (Hitachi, Tokyo, Japan). From each of the experimental groups, the kidney tissues were fixed with 4% paraformaldehyde (pH 7.4) then embedded in paraffin. Two-micrometer tissue sections were processed for analysis. Standard protocols were used to stain these tissue sections with periodic acid-Schiff and Masson’s trichrome to evaluate histological changes and collagen deposition, respectively. Regions of collagen deposition in Masson’s trichrome stained kidney sections were measured using an image analysis program (i-solution; Image & Microscope Technology Inc., Daejeon, South Korea).

### 4.4. Immunohistochemistry

Immunohistochemical staining was performed using by anti-fibronectin (1:100; Abcam, Cambridge, UK) antibodies, anti-α-SMA (1:100; Abcam, Cambridge, UK), anti-osteopontin (OPN) (1:1000; MPIIIB10, obtained from the Developmental Studies Hybridoma Bank, University of Iowa, Iowa City, IA, USA), and anti-ED-1 (1:100; Abcam, Cambridge, UK). EnVision-HRP kit (Dako, Carpinteria, CA, USA) was used for detection. Counterstaining of all the sections was performed using Mayer’s hematoxylin. A Leica DM IRB inverted microscope (Leica Microsystems, Wetzlar, Germany) equipped with a CoolSNAP HQ camera (Photometrics, Tucson, AZ, USA) was used to examine immunolabeling. The quantitation of fibronectin and ED-1 positive area was measured with an image analysis program (i-solution, Image & Microscope Technology Inc., Daejeon, South Korea).

### 4.5. Terminal Deoxynucleotidyl Transferase-Mediated dUTP Nick-End Labeling (TUNEL) Assay

The TUNEL assay was performed using the Click-iTTM TUNEL colorimetric IHC Detection Kit (Life Technologies, Carlsbad, CA, USA) for immunohistochemistry as per the manufacturer’s protocol. The number of TUNEL-positive cells was counted in 10 fields at 400× magnification and normalized per mm2 of tissue.

### 4.6. Measurement of Caspase-3 Activity

We used a colorimetric assay kit (Sigma-Aldrich, St. Louis, MO, USA) to measure caspase-3 activity in the kidney homogenates as per the manufacturer’s protocol. The kidney homogenates were incubated with fluorometric caspase-3 substrate (Ac-DEVD-pNA) in assay buffer. A control reaction mixture containing the caspase-3 inhibitor (acetyl-DEVD-CHO) in assay buffer was used in order to explain nonspecific hydrolysis of the substrate. The two mixtures were incubated at 37 °C for 90 min, and the absorbance was measured at 405 nm.

### 4.7. Western Blotting Assay

Immunoblotting using kidney tissues was performed with primary antibodies against α-SMA (1:5000; Sigma-Aldrich), Bcl-2 (1:1000; Cell Signaling Technology, Danvers, MA, USA), and Bax (1:1000; Cell Signaling Technology). Antigen−antibody reaction sites were detected with horseradish peroxidase conjugated secondary antibodies (1:2000; Dako, Glostup, Denmark) using an enhanced chemiluminescence (ECL) advanced detection system (GE Healthcare, Little Chalfont, UK). We quantified the band densities using densitometry and compared those to the expression of glyceraldehyde-3-phosphate dehydrogenase (GAPDH). The band pixel intensities were analyzed by computerized method performed by Scion Image (Scion, Frederick, MD, USA).

### 4.8. Statistical Analysis

Data are presented as means ± standard error of the mean values. Experiments were repeated at least thrice. Statistical analyses were performed using Kruskal–Wallis test, followed by post hoc Mann–Whitney U test with Bonferroni correction. The analyses were performed with SPSS (version 20.0, IBM Corp., Armonk, NY, USA). *p* < 0.05 was considered to indicate statistical significance.

## Figures and Tables

**Table 1:** Basic parameters.

	VH (*n* = 6)	AAT (*n* = 6)	TAC (*n* = 6)	TAC + AAT (*n* = 6)
Baseline BW, g	254.3 ± 5.0	253.0 ± 7.0	253.0 ± 5.2	256.7 ± 5.5
Last BW, g	400.3 ± 8.9	397.7 ± 14.7	364.3 ± 6.4 ***	358.7 ± 12.8 ***
BW gain, g	146.0 ± 10.8	144.7 ± 18.4	111.3 ± 7.2 ***	102.0 ± 14.4 ***
Serum BUN, mg/dL	16.1 ± 1.0	15.5 ± 1.4	61.1 ± 8.9 ***	45.0 ± 4.8 ***^, ##^
Serum creatinine, mg/dL	0.40 ± 0.04	0.39 ± 0.03	0.67 ± 0.11 ***	0.53 ± 0.05 ***^, ##^
TAC con, ng/mL	NA	NA	26.3 ± 4.8	25.7 ± 5.1

Values are presented as mean ± SD; *** *p* < 0.001 vs. VH; ^##^ *p* < 0.01 vs. TAC. Abbreviations: VH, vehicle group, AAT, alpha-1 antitrypsin treatment group; TAC, tacrolimus treatment group; TAC + AAT, tacrolimus and alpha-1 antitrypsin cotreatment group; BW, body weight; BUN, blood urea nitrogen; TAC con, tacrolimus concentration.
